# Clinically relevant germline variants in allogeneic hematopoietic stem cell transplant recipients

**DOI:** 10.1038/s41409-022-01828-x

**Published:** 2022-10-04

**Authors:** Atte K. Lahtinen, Jessica Koski, Jarmo Ritari, Kati Hyvärinen, Satu Koskela, Jukka Partanen, Kim Vettenranta, Minna Koskenvuo, Riitta Niittyvuopio, Urpu Salmenniemi, Maija Itälä-Remes, Kirsi Jahnukainen, Outi Kilpivaara, Ulla Wartiovaara-Kautto

**Affiliations:** 1 Applied Tumor Genomics Research Program, Faculty of Medicine, University of Helsinki, Helsinki, Finland; 2 Department of Medical and Clinical Genetics, Medicum, Faculty of Medicine, University of Helsinki, Helsinki, Finland; 3 Research and Development, Finnish Red Cross Blood Service, Helsinki, Finland; 4 New Children’s Hospital, Pediatric Research Center, University of Helsinki and Helsinki University Hospital, Helsinki, Finland; 5 Helsinki University Hospital, Comprehensive Cancer Center, Department of Hematology, and University of Helsinki, Helsinki, Finland; 6 Turku University Hospital, Department of Clinical Hematology and Stem Cell Transplant Unit, and University of Turku, Turku, Finland; 7 NORDFERTIL Research Lab Stockholm, Department of Women’s and Children’s Health, Karolinska Institutet and University Hospital, Stockholm, Sweden; 8 HUSLAB Laboratory of Genetics, HUS Diagnostic Center, Helsinki University Hospital, Helsinki, Finland

**Keywords:** Cancer genetics, Genetics research, Cancer genetics, Haematological cancer

## Abstract

Allogeneic hematopoietic stem cell transplantation (HSCT) provides patients with severe hematologic disease a well-established potential for curation. Incorporation of germline analyses in the workup of HSCT patients is not a common practice. Recognizing rare harmful germline variants may however affect patients’ pre-transplantation care, choice of the stem cell donor, and complication risks. We analyzed a population-based series of germline exome data of 432 patients who had undergone HSCT. Our aim was to identify clinically relevant variants that may challenge the outcome of the HSCT. We focused on genes predisposing to hematological diseases, or solid tumors, and genes included in the American College of Medical Genetics secondary findings list v3.0. As population-specific controls, we used GnomAD non-cancer Finns (*n* = 10,816). We identified in our population-based analysis rare harmful germline variants in disease-predisposing or actionable toxicity-increasing genes in 17.8% of adult and pediatric patients that have undergone HSCT (15.1% and 22.9%, respectively). More than half of the patients with a family member as a donor had not received genetic diagnosis prior to the HSCT. Our results encourage clinicians to incorporate germline genetic testing in the HSCT protocol in the future in order to reach optimal long-term outcome for the patients.

## Introduction

Despite recent progress in cellular and genetic therapy modalities for blood diseases, allogeneic hematopoietic stem cell transplantation (HSCT) remains the major curative treatment for high-risk hematological malignancies, inborn errors of immunity, and hypoplastic bone marrow syndromes. The HSCT protocol has been optimized for decades. Still today, new aspects arise and precision medicine, including germline genetics, will likely produce novel cues.

Recognizing germline defects may require modified pre-transplant strategies: it affects the selection of the donor and determines the appropriate conditioning [1]. This practice is well-acknowledged in the treatment of inherited bone marrow failure (BMF) syndromes, like Fanconi anemia and short telomere disorders [2, 3]. However, the increasing amount of information on the role of germline defects in hematological diseases challenges us to think more broadly [4, 5]. Allogeneic HSCT itself predisposes patients to secondary tumors. Furthermore, certain germline-derived BMFs demonstrate high risk for subsequent malignancy or organ dysfunction after HSCT [6–8].

We are witnessing remarkable improvement in the primary therapy results of hematological diseases. Particularly in young adults and pediatric patients, we need to address the long-term effects and complications of therapy where germline genetics will have a role.

We analyzed a population-based set of germline exome data of 432 adult and pediatric hematologic patients that have undergone allogeneic HSCT in 1999-2020. Our aim was to determine the prevalence of clinically relevant, rare pathogenic (P) and likely pathogenic (LP), germline variants in genes predisposing an individual to hematological malignancies, solid tumors, and other comorbidities potentially hampering the success of HSCT.

## Materials and methods

### Patients

In Finland, HSCTs are centralized in Helsinki University Hospital (HUH; adult and pediatric patients) and Turku University Hospital (TUH; adults). We analyzed whole exome sequencing (WES) data in three patient cohorts: 141 adult patients from HUH and TUH (Adult cohort 1, patients D001–D141), 138 adult patients from the Finnish Bone Marrow Transplantation Registry (BMTR) (Adult cohort 2, patients V001–V138), and 153 pediatric patients from HUH (Pediatric cohort, patients C001–C153). Study patients’ clinical information was gathered from medical records and the Finnish Hematological Registry (adults). The patient characteristics are summarized in Table 1 and Supplementary Table 1. The study was approved by the Ethics Committees of HUH and TUH, and the Finnish National Supervisory Authority for Welfare and Health (Valvira) (#206/13/03/03/2016, #303/13/03/01/2011; HUS/114/2018, HUS/284/2019, and V/3235/2019). Samples and data were collected either after written informed consent (living individuals), or authorization by the ethics committee (deceased patients).

**Table 1:** Patient characteristics.

Characteristic	Adult cohort 1	Adult cohort 2	Pediatric cohort
Age at HSCT (years)	17–70	16–69	0–18
Mean	45.8	49.1	8.7
Median	48.2	52.0	8.2
Number of patients	141	138	153
Male	69 (49%)	68 (50%)	102 (67%)
Female	72 (51%)	68 (50%)	51 (33%)
NA	0	2	0
Diagnoses			
Myeloid malignancies (AML, MDS, MPN)	97	78	35
ALL	29	22	88
Plasma cell dyscrasias	12	21	0
AA/BMF	3	8	11
PID	0	0	8
Other^a^	0	9	11
Source of transplant (MUD/SIB/haplo)	111/26/4	0/138/0	86/64/3
Type of transplant (PB/BM/cord/NA)	121/19/1/0	101/33/0/4	0/130/23/0
Conditioning (MAC/RIC/NA)	118/23/0	93/43/2	143/10/0

*AA* aplastic anemia, *ALL*  acute lymphoblastic leukemia, *AML*acute myeloid leukemia, *BM* bone marrow, *BMF* bone marrow failure, *MAC* myeloablative conditioning, *MDS*myelodysplastic syndrome, *MPN*myeloproliferative neoplasm, *MUD* matched unrelated donor, *NA* not applicable, *PB* peripheral blood, *PID* primary immunodeficiency, *RIC* reduced-intensity conditioning, *SIB* Sibling.

^a^Other diagnoses included eight chronic lymphocytic leukemias, and one prolymphocytic leukemia in adult patients; four non-Hodgkin lymphomas, two Langerhans cell histiocytosis, two solid tumor malignancies, one adrenoleukodystrophy, one betathalassemia, and one osteopetrosis in pediatric patients.

### Adult cohort 1

The adult cohort 1 included patients with allogeneic HSCT performed in 1999–2019 (1999–2011 *n* = 16; 2012–2019 *n* = 125) in HUH (*n* = 134) or TUH (*n* = 7). Patients were 17–70 years of age (mean 45.8 years, median 48.2 years) at the time of HSCT (Table 1). DNA for exome sequencing was extracted from skin biopsies (*n* = 138), bone marrow (*n* = 2; D058 and D092) or peripheral blood (*n* = 1; D024). Samples were originally collected as germline controls for somatic (bone marrow or peripheral blood) exome analysis for academic research purposes [9, 10] or retrieved from the Finnish Hematological Registry and Biobank. Exome sequencing and bioinformatics have been described earlier [11].

### Adult cohort 2

The adult cohort 2 consisted of adult patients from the Finnish BMTR originally described in Ritari et al. [12]. These data collection consisted of patients that had undergone HSCT from an HLA-matched sibling donor between years 2002 and 2016 in Finland (HUH, *n* = 47; TUH *n* = 91). Exome sequencing data were available for 163 patients. We excluded three subjects already included in the adult cohort 1, five pediatric patients, and 17 adult patients with solid cancer as the indication for HSCT. Patients were between 16 and 69 years of age (mean 49.1 years, median 52.0 years) at the time of HSCT (Table 1). DNA for exome sequencing was extracted from blood samples originally collected or HLA typing before transplantation. The exome sequencing and bioinformatics are described in detail in Morin et al. [13]. This adult cohort 2 is a population-based series of allogeneic transplant recipients and matches the adult cohort 1 for age, gender, and diagnosis (*p* = 0.072, *p* = 0.86, *p* = 0.071, respectively: Mann-Whitney test) (Table 1).

### Pediatric cohort

To compare frequencies of germline variants among the adult transplant recipients, we also analyzed a data set comprising all children who had undergone HSCT in Finland 2001–2020 (*n* = 352) and for whom exome data (performed for academic research purposes) was available (*n* = 153, 43.5%). DNA for exome sequencing was extracted from blood samples collected for HLA typing before transplantation. The sample preparation and exome sequencing have been previously described [11]. Pediatric patients were 0–18 years of age (mean 8.7 years, median 8.2 years) at the time of HSCT. The major indication for HSCT was acute lymphoblastic leukemia (ALL), but the set also included some other rare malignant and non-malignant diseases (Table 1 and Supplementary Table 1). We compared clinical characteristics of the patients with and without exome data: Age, gender, and diagnosis distribution of the patients did not differ between these patient series (data not shown), and the pediatric cohort represents population-based pediatric material.

### Genes included in the analyses

We used three gene lists to study clinically relevant rare germline variants in our study patients. First, we gathered a list of 189 genes predisposing to hematological malignancies, cytopenia syndromes, and inborn errors of immunity (*Hematology Panel*, Supplementary Table 2). Second, we formatted a list of 114 genes predisposing patients to solid cancers (*Oncology Panel*, Supplementary Table 2). Third, we used a list of 73 clinically actionable genes that the American College of Medical Genetics and Genomics (ACMG) has compiled (*ACMG SF 3.0 Panel*, Supplementary Table 2) [14]. The genes in each panel shared features, e.g. risk of graft failure or increased risk of secondary malignancy, and were thus analyzed together. Deleterious variants in the *ACMG SF 3.0 Panel* genes are known to cause disorders that have clinical guidelines for intervention.

### Variant analyses

We analyzed the variants utilizing next-generation sequencing data with BasePlayer (1.0.2) [15]. We validated the findings visually with BasePlayer and the somatic exomes were utilized to validate the findings in the adult cohort 1. We used GnomAD non-cancer whole database and GnomAD non-cancer Finns (*n* = 10,816) as population-specific controls (version 2.1) [16, 17]. Variants with a minor allele frequency higher than 0.05 were not considered. To filter out poor-quality variants we used a 1000 genomes mappability pilot mask track [18] and quality measures of genotype quality ≥ 20, QUAL ≥ 20, coverage > 6 reads, and allelic fraction ≥ 30%.

We classified the variants as benign, likely benign, variant of uncertain significance, LP, and P according to the ACMG guidelines [19] using two different variant classification tools: Varsome [20] (ACMG classification, version 10.1.1–10.2.3) and Intervar [21] (version 2.0.2). In addition, ClinVar interpretations [22] (version 2016-03-02) annotated by Intervar were utilized to assess the pathogenicity of the variants.

The blood samples (adult cohort 2 and pediatric cohort) for HLA typing and exome sequencing were obtained at different phases of the disease. We filtered somatic mutations by considering the variant allele frequency (VAF), characteristics of the variant, and features of the patient’s disease (blast count in acute leukemias). Some variants with VAF > 0.5 were classified as heterozygous after considering the variant type, sequencing depth, sample type, and patient’s disease status. When the evaluation was ambiguous, the variant was conservatively classified as heterozygous to avoid overestimation. In addition, we used gnomAD database (germline variants) and the Catalogue of somatic mutations in cancer to evaluate the origin of the variant.

We report variants with at least two P or LP predictions, and variants that are previously evaluated in ClinVar as P/LP or in another undisputable reference (#D028, #D049, #D063, #C071), and in two cases with strong genotype-phenotype correlation (#C047, #C113), being conservative in our interpretation (Supplementary Table 3). In our results, we only report patients carrying biallelic variants in genes causing an autosomal recessive disorder or monoallelic variants in genes causing an autosomal dominantly inherited or X-linked disorder.

## Results

**Fig. 1:**
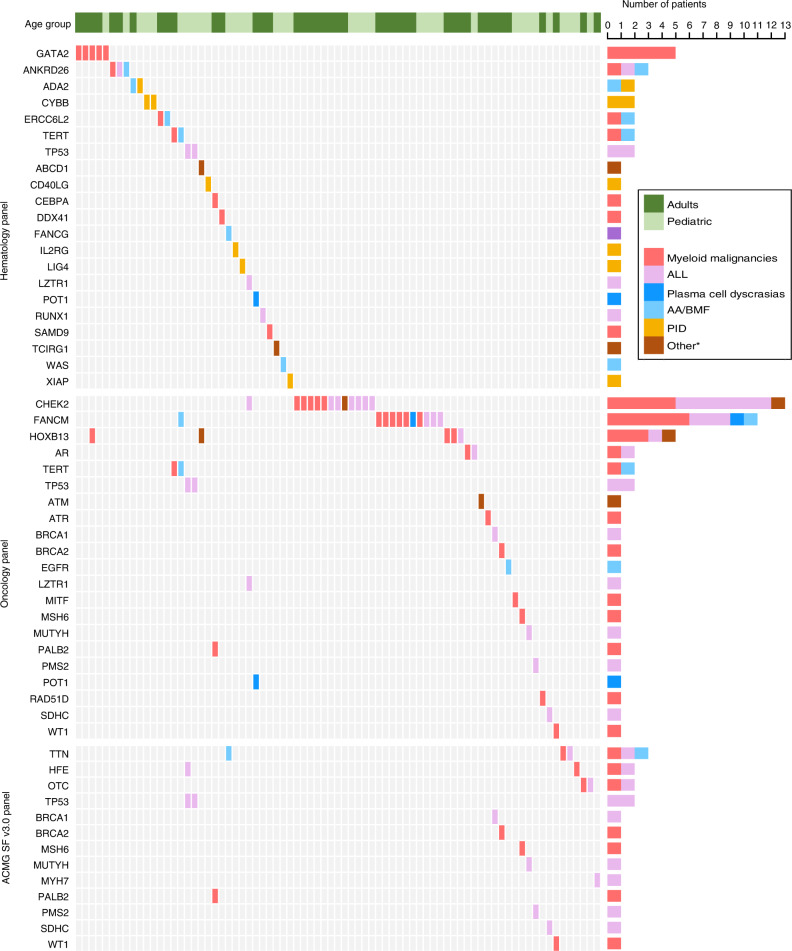
Visualizing variants as an oncoplot. Each row represents a gene and each column a patient with one or more rare harmful germline variants. Number of patients with a variant in each gene is demonstrated in the bar plot on the right side. * Other diagnoses include chronic lymphocytic leukemia in adults and osteopetrosis and adrenoleukodystrophy in pediatric patients. ALL – Acute lymphoblastic leukemia, AA – Aplastic anemia, BMF – Bone marrow failure, PID – Primary immunodeficiency.

In our study, of the 432 transplant recipients, 17.8% (42/279, 15.1% of adult patients combined from the adult cohorts 1 and 2; 35/153, 22.9% of pediatric patients) carried a pathogenic (P) or likely pathogenic (LP) variant in at least one of the genes analyzed. The oncoplot demonstrates all genes in which P/LP variants were discovered (Fig. 1). Rare harmful germline variants in *CHEK2* (13 patients), and *FANCM* (11 patients), *HOXB13* (five patients), *GATA2* (five patients), and *ANKRD26* (three patients) were the most prevalent.

The number of adult patients with clinically relevant germline variants in the *Hematology Panel* was comparable (*p* = 0.289) in the adult cohorts 1 and 2 (10/141, 7.1%, and 5/138, 3.6%, respectively), (Table 2). Of the adult patients with ALL or myeloid malignancy, 6.3% (8/126, adult cohort 1) and 4.0% (4/100, adult cohort 2) had P/LP variants in well-known predisposing genes for these diseases. Also, other diagnostic groups included patients with deleterious variants but the total number of patients in these groups was too limited for further analyses. There was no difference in overall survival between patients with and without harmful germline variants. The analysis was restricted to the largest patient group, adult AML patients with survival data available (*n* = 65, Supplementary Fig. 1). In comparison to the pediatric patients (11.1%, 17/153), the proportion of adults (5.4%, 15/279) with P/LP variants in the *Hematology Panel* genes was significantly lower (*p* = 0.035), (Table 2). The difference is explained by the high frequency of germline mutations in severe childhood syndromes (AA/BMF and primary immune deficiencies (PID)). Five out of ten patients with germline variants in the adult cohort 1 and 11/17 of the pediatric cohort had a genetic diagnosis before HSCT (data not available for the adult cohort 2) (Supplementary Table 3).

**Table 2:** Proportion of patients with clinically relevant rare variants in HSCT recipients.

Gene Panel/Diagnosis group	Adult cohort 1	Adult cohort 2	Total (adults)	Pediatric cohort	Adults vs. children *p* value^b^
All Panels	24/141 (17.0%)	18/138 (13.0%)	42/279 (15.1%)	35/153 (22.9%)	0.049
Myeloid malignancies (AML, MDS, MPN)	17/97 (17.5%)	11/78 (14.1%)	28/175 (16.0%)	6/35 (17.1%)	0.806
ALL	4/29 (13.8%)	3/22 (13.6%)	7/51 (13.7%)	16/88 (18.2%)	0.637
Plasma cell dyscrasias	1/12 (8.3%)	1/21 (4.8%)	2/33 (6.1%)	-	NA
AA/BMF	2/3 (66.7%)	1/8 (12.5%)	3/11 (27.3%)	4/11 (36.4%)	1.000
PID	-	-	-	7/8 (87.5%)	NA
Other^a^	-	2/9 (22.2%)	2/9 (22.2%)	2/11 (18.2%)	NA
Hematology Panel	10/141 (7.1%)	5/138 (3.6%)	15/279 (5.4%)	17/153 (11.1%)	0.035
Myeloid malignancies (AML, MDS, MPN)	7/97 (7.2%)	3/78 (3.8%)	10/175 (5.7%)	1/35 (2.9%)	0.695
ALL	1/29 (3.4%)	1/22 (4.5%)	2/51 (3.9%)	3/88 (3.4%)	1.000
Plasma cell dyscrasias	1/12 (8.3%)	0/21 (0.0%)	1/33 (3.0%)	-	NA
AA/BMF	1/3 (33.3%)	1/8 (12.5%)	2/11 (18.2%)	4/11 (36.4%)	0.635
PID	-	-	-	7/8 (87.5%)	NA
Other^a^	-	0/9 (0.0%)	0/9 (0.0%)	2/11 (18.2%)	NA
Oncology Panel	18/141 (12.8%)	11/138 (8.0%)	29/279 (10.4%)	19/153 (12.4%)	0.526
Myeloid malignancies (AML, MDS, MPN)	13/97 (13.4%)	7/78 (9.0%)	20/175 (11.4%)	3/35 (8.6%)	0.773
ALL	3/29 (10.3%)	1/22 (4.5%)	4/51 (7.8%)	14/88 (15.9%)	0.200
Plasma cell dyscrasias	1/12 (8.3%)	1/21 (4.8%)	2/33 (6.1%)	-	NA
AA/BMF	1/3 (33.3%)	0/8 (0.0%)	1/11 (9.1%)	1/11 (9.1%)	1.000
PID	-	-	-	0/8 (0.0%)	NA
Other^a^	-	2/9 (22.2%)	2/9 (22.2%)	1/11 (9.1%)	NA
ACMG SF v3.0 Panel	3/141 (2.1%)	3/138 (2.2%)	6/279 (2.2%)	11/153 (7.2%)	0.017
Myeloid malignancies	2/97 (2.1%)	2/78 (2.6%)	4/175 (2.3%)	3/35 (8.6%)	0.092
ALL	1/29 (3.4%)	1/22 (4.5%)	2/51 (3.9%)	7/88 (8.0%)	0.486
Plasma cell dyscrasias	0/12 (0.0%)	0/21 (0.0%)	0/33 (0.0%)	-	NA
AA/BMF	0/3 (0.0%)	0/8 (0.0%)	0/11 (0.0%)	1/11 (9.1%)	1.000
PID	-	-	-	0/8 (0.0%)	NA
Other^a^	-	0/9 (0.0%)	0/9 (0.0%)	0/11 (0.0%)	NA

There was no significant difference between the two adult patient groups in any of the subgroups. Pediatric patients had more variants detected in the Hematology Panel and ACMG SF v3.0 Panel.

*AA* aplastic anemia, *ALL* acute lymphoblastic leukemia, *AML* acute myeloid leukemia, *BMF* bone marrow failure, *MDS* myelodysplastic syndrome, *MPN* myeloproliferative neoplasm, *NA* not applicable, *PID* primary immunodeficiency.

^a^Other diagnoses included eight chronic lymphocytic leukemias and one prolymphocytic leukemia in adult patients and four non-Hodgkin lymphomas, two Langerhans cell histiocytosis, two solid tumor malignancies, one adrenoleukodystrophy, one betathalassemia, and one osteopetrosis in pediatric patients.

^b^Fisher’s exact test.

We detected rare P/LP germline variants in the *Oncology panel* genes in similar proportions in all study sets: 12.8% (18/141), 8.0% (11/138), and 12.4% (19/154) in the adult cohort 1, adult cohort 2, and pediatric cohort, respectively (adult cohort 1 vs. 2 *p* = 0.240; adult patients vs. pediatric patients *p* = 0.526). There were no significant differences in the carrier frequencies between the various diagnosis groups (Table 2).

The ACMG Secondary Finding (SF) panel consists of both cancer and other severe disease predisposing genes [14]. In our patient series, 11 children and six adults had a P/LP variant in these genes (Table 2). Notably, 10/17 of the variants were in genes also included in the *Oncology Panel*, and one adult and four children had other than a malignancy predisposing gene defect (Supplementary Table 3).

## Discussion

Germline genetic testing is becoming increasingly accessible and should be considered when performing such a resource-demanding therapeutic process as HSCT. Our population-based analysis shows rare harmful germline variants in well-established disease-causing or actionable toxicity-increasing genes in 13–23% of adult and pediatric patients that have undergone HSCT (Table 2). This accentuates the need for precision medicine covering not only somatic, but also germline genetics when striving for an optimal HSCT survivorship.

The frequencies of the P/LP variants in our set of 432 patients were as expected and in line with the estimates derived from studies published on hematological patients, but without a focus on HSCT (Table 2) [5, 23–25]. Predictably, variants in the *Hematology Panel* were more prevalent in pediatric patients than in adults, as most of the genes in this list have originally been discovered in children (and families) with a hematological disease or PID and categorized as high penetrance genes [23, 25].

The identification of a predisposing gene variant in hematologic patients has implications when planning HSCT: It is crucial to refrain from employing an affected family member or an asymptomatic carrier as a donor. However, when no evidence or suspicion of inherited disease, we should be conservative with the screening of healthy donors, especially with unrelated donors. Incorporating germline genetics in the patients’ clinical workup preceding HSCT may also give an opening for reproductive and genetic counseling, and follow-up of at-risk family members [26].

In our study, 50–65% of the P/LP variants in Hematology Panel genes were identified by routine measures preceding HSCT (5/10 in adult patients and 11/17 in pediatric patients, data not available for the adult cohort 2; Supplementary Table 3). In contrast, clinically relevant findings *not* recognized promptly were e.g., in *TP53*, *TERT*, and *RUNX1*. We detected rare harmful variants in any of the panel genes in 22/59 of the HSCT patients with a family donor (37.3%) in the adult cohort 1 and pediatric cohort. We consider this finding striking. In Finland, registry donors are over-represented compared to many other HSCT centers due to HLA haplotype distribution in the Finnish population and good registry donor availability [27]. This was also reflected in the donor distribution in this study and implies that the risk is even higher in centers using predominantly family donor strategies. Success of alternative donor HSCT strategies among patients who lack fully HLA-matched donors will further increase the use of family donors in the future.

An equal number of adults and children had a P/LP variant in the *Oncology panel* genes (Table 2). This may imply that deleterious variants in genes which predispose only to solid tumors would rarely cause a disease that would indicate HSCT. Pre-transplant treatment exposure, conditioning regimen, and transplant-related complications are associated with a wide range of late adverse effects that deleterious germline variants may increase. This is important for optimal screening, diagnosis, and treatment of the patient. Solid tumor predisposing genes are not routinely analyzed prior to HSCT. However, combined with an increased risk caused by a genotoxic assault, genetic factors may expose patients to immoderate risk for secondary malignancies [6, 28]. Our study set included patients with both high and moderate penetrance solid cancer risk variants in DNA repair genes, e.g., *TP53*, *BRCA1*, *BRCA2*, *CHEK2*, and *FANCM*. To improve long-term survival, these patients require individualized cancer surveillance plan as part of the HSCT follow-up, and in some cases tailoring of the conditioning modalities. Moreover, we have only little information on the combined toxicity burden linked to e.g., DNA repair defects and hematological malignancy treatment before HSCT. Nevertheless, based on studies on (non-malignant) inherited BMF syndromes, we can estimate that the toxicity risk is increased [29–31].

CHEK2 and FANCM are DNA repair proteins, and deleterious variants in these genes are well-established for solid tumor predisposition [32, 33]. In our study, we observed several variants in these genes. However, when compared to our population-matched controls, they were as common in a normal cancer-free population. This highlights the value of a good control set, such as GnomAD Finns. Conversely, the normal population will never encounter the genotoxic stress introduced by HSCT. Yet, these variants may nevertheless contribute to toxicities or secondary cancers and should thus not be neglected.

When recommending genetic tests, patients consented to both a disease-specific gene analysis and for incidental findings (*ACMG SF 3.0*). These genes and their significance are very well documented and intervention guidelines exist. Acknowledging the ACMG SF genes would improve the likelihood of a successful HSCT. The range of P/LP variants in our patient sets correlates well with published data [34]. It is not surprising that children harbored more variants in the *ACMG SF 3.0* genes since mutations in many of these cause severe diseases. It is possible that individuals harboring them may have succumbed before adulthood or symptomatic disease has hampered performing HSCT. How these gene variants affect the short- and long-term tolerance of HSCT, remains to be evaluated. Late toxicities may surface, e.g. with underlying hemochromatosis, as was the case in two children with homozygous *HFE* mutations in the pediatric cohort (Supplementary Table 3).

The study centers HUH and TUH are the only units performing HSCTs in Finland. Our data represent ~15% of randomly selected adult and 45% of pediatric transplant recipients. As healthcare in Finland is complimentary, the patients’ socioeconomic status has no significant effect on the access to HSCT if medically indicated, which further supports the representativity of the data.

We acknowledge the restriction in the number of our study patients representing various disease groups, and the lack of performing a copy number analysis on the exome data. However, our aim was to define the minimum number of clinically relevant germline defects, and not to search for new candidate variants or alterations. We only accounted for variants with confirmed pathogenicity and thus may have missed variants for which the significance will be established in the future. We were not either able to show difference in overall survival of patients with and without harmful germline variants. This may be due to limited number of patients or restricted follow-up period. Despite the limited number of study patients, the whole population is well represented. Cancer incidence in Finland is comparable to other developed countries, therefore our results are more widely applicable.

Personalized medicine is the future of all cancer treatment. Today, hematology still heavily leans on HSCT, which causes high systemic toxicity burden and involves the donor’s genetics, as the major curative approach. Hence, hematological diseases should be at the forefront of this development. We propose timely screening of all transplant recipients for well-documented rare harmful germline defects. This improves the chances of healthy donor selection, offers variant-specific counseling, and enables life-long vigilance for follow-up of secondary malignancies and other adverse effects among affected recipients.

## Supplementary information

Supplemental Data

## Data Availability

The data generated and analyzed in the study are not publicly available due to privacy and ethical restrictions but are available from the corresponding author upon reasonable request.
