# Supplementary material for: Clinically relevant germline variants in allogeneic hematopoietic stem cell transplant recipients
Source: Bone Marrow Transplant. 2022 Oct 4;58(1):39–45. doi: 10.1038/s41409-022-01828-x (PMC9812774; doi:10.1038/s41409-022-01828-x)
Supplement: Supplementary file 1 — Supplemental Data [file 41409_2022_1828_MOESM1_ESM.docx]

Supplemental Data

Table of Contents

[Supplemental Table 1. Distribution of diagnoses 2](#_Toc107699213)

[Supplemental Table 2. Gene panels 3](#_Toc107699214)

[Supplemental Table 2A. Hematology Panel 3](#_Toc107699215)

[Supplemental Table 2B. Oncology Panel 6](#_Toc107699216)

[Supplemental Table 2C. ACMG SF v3.0 Panel 9](#_Toc107699217)

[Supplemental Table 3. Harmful variants in analyzed genes 11](#_Toc107699218)

[Supplemental Table 3A. Adult cohort 1 11](#_Toc107699219)

[Supplemental Table 3B. Adult cohort 2 13](#_Toc107699220)

[Supplemental Table 3C. Pediatric cohort 14](#_Toc107699221)

[Supplemental Figure 1. Kaplan-Meier survival curves for adult AML patients 16](#_Toc107699222)

[Supplemental Figure 1A. Hematology Panel 16](#_Toc107699223)

[Supplemental Figure 1B. Oncology Panel 16](#_Toc107699224)

[Supplemental Figure 1C. ACMG SF v3.0 Panel 17](#_Toc107699225)

[Supplemental Figure 1D. All Panels 17](#_Toc107699226)

[References 18](#_Toc107699227)

Supplemental Table 1. Distribution of diagnoses, n (%). *Primary immunodeficiencies include three chronic granulomatous diseases (CGD), two severe combined immunodeficiencies (SCID), one hyper-IgM syndrome, one adenosine deaminase 2 (ADA2) deficiency, and one lymphoproliferative syndrome (XLP2)

| **Diagnoses** | **Adult cohort 1** | **Adult cohort 2** | **Pediatric cohort** |
| --- | --- | --- | --- |
| Acute myeloid leukemia | 68 (48) | 54 (39) | 22 (14) |
| Acute lymphocytic leukemia | 29 (21) | 22 (16) | 88 (58) |
| Multiple myeloma | 12 (9) | 20 (14) | - |
| Myelodysplastic syndrome | 14 (10) | 10 (7) | 4 (3) |
| Chronic myeloid leukemia | 8 (6) | 3 (2) | 6 (4) |
| Aplastic anemia | 2 (1) | 8 (6) | 9 (6) |
| Chronic lymphocytic leukemia | - | 8 (6) | - |
| Chronic myelomonocytic leukemia | 4 (3) | 3 (2) | - |
| Mixed phenotype acute leukemia | 2 (1) | 3 (2) | - |
| Myelofibrosis | 1 (1) | 4 (3) | - |
| Bone marrow failure | 1 (1) | - | 2 (1) |
| Mastocytosis | - | 1 (1) | - |
| Plasma cell leukemia | - | 1 (1) | - |
| Prolymphocytic leukemia | - | 1 (1) | - |
| Primary immunodeficiency* | - | - | 8 (5) |
| Non-Hodgkin lymphoma | - | - | 4 (3) |
| Juvenile myelomonocytic leukemia | - | - | 3 (2) |
| Langerhans cell histiocytosis | - | - | 2 (1) |
| Solid tumor | - | - | 2 (1) |
| Adrenoleukodystrophy | - | - | 1 (1) |
| Betathalassemia | - | - | 1 (1) |
| Osteopetrosis | - | - | 1 (1) |

Supplemental Table 2. Gene panels**.** a) Hematology Panel, b) Oncology Panel, and c) ACMG SF v3.0 Panel. AD - Autosomal dominant, AR - Autosomal recessive, XLD - X-linked dominant, XLR - X-linked recessive.

## Supplemental Table 2A. Hematology Panel

| **Gene** | **Inheritance** | **Syndrome** |
| --- | --- | --- |
| ABCD1 | XLR | Adrenoleukodystrophy, X-linked |
| ATRX | XLD | Alpha-thalassemia/mental retardation syndrome |
| SAMD9L | AD | Ataxia-pancytopenia syndrome, Monosomy 7 myelodysplasia and leukemia syndrome |
| ATM | AR | Ataxia-telangiectasia |
| CASP10 | AD | Autoimmune lymphoproliferative syndrome |
| CTLA4 | AD | Autoimmune lymphoproliferative syndrome |
| FAS | AD | Autoimmune lymphoproliferative syndrome |
| FASLG | AD | Autoimmune lymphoproliferative syndrome |
| PRKCD | AR | Autoimmune lymphoproliferative syndrome |
| TAZ | XLR | Barth syndrome |
| BLM | AR | Bloom syndrome |
| DNAJC21 | AR | Bone marrow failure syndrome |
| ERCC6L2 | AR | Bone marrow failure syndrome |
| SRP72 | AD | Bone marrow failure syndrome |
| MAP2K1 | AD | Cardiofaciocutaneous syndrome |
| MAP2K2 | AD | Cardiofaciocutaneous syndrome |
| RMRP | AR | Cartilage-hair hypoplasia |
| CTC1 | AR | Cerebroretinal microangiopathy with calcifications and cysts |
| STN1 (OBFC1) | AR | Cerebroretinal microangiopathy with calcifications and cysts |
| LYST | AR | Chediak-Higashi syndrome |
| CYBA | AR | Chronic granulomatous disease |
| CYBC1 (C17orf62) | AR | Chronic granulomatous disease |
| NCF1 | AR | Chronic granulomatous disease |
| NCF2 | AR | Chronic granulomatous disease |
| NCF4 | AR | Chronic granulomatous disease |
| CYBB | XLR | Chronic granulomatous disease, X-linked |
| MBD4 | AR | Clonal hematopoiesis and early-onset AML |
| CLPB | AR | CLPB deficiency |
| VPS13B | AR | Cohen syndrome |
| MTHFD1 | AR | Combined immunodeficiency and megaloblastic anemia with or without hyperhomocysteinemia |
| IKZF1 | AD | Common variable immunodeficiency |
| C15ORF41 | AR | Congenital dyserythropoietic anemia |
| CDAN1 | AR | Congenital dyserythropoietic anemia |
| KIF23 | AD | Congenital dyserythropoietic anemia |
| KLF1 | AD | Congenital dyserythropoietic anemia |
| SEC23B | AR | Congenital dyserythropoietic anemia |
| HRAS | AD | Costello syndrome |
| GATA1 | XLR | Diamond-Blackfan anemia |
| RPL11 | AD | Diamond-Blackfan anemia |
| RPL15 | AD | Diamond-Blackfan anemia |
| RPL18 | AD | Diamond-Blackfan anemia |
| RPL26 | AD | Diamond-Blackfan anemia |
| RPL27 | AD | Diamond-Blackfan anemia |
| RPL31 | AD | Diamond-Blackfan anemia |
| RPL35 | AD | Diamond-Blackfan anemia |
| RPL35A | AD | Diamond-Blackfan anemia |
| RPL36 | AD | Diamond-Blackfan anemia |
| RPL5 | AD | Diamond-Blackfan anemia |
| RPL9 | AD | Diamond-Blackfan anemia |
| RPS10 | AD | Diamond-Blackfan anemia |
| RPS15 | AD | Diamond-Blackfan anemia |
| RPS15A | AD | Diamond-Blackfan anemia |
| RPS17 | AD | Diamond-Blackfan anemia |
| RPS19 | AD | Diamond-Blackfan anemia |
| RPS24 | AD | Diamond-Blackfan anemia |
| RPS26 | AD | Diamond-Blackfan anemia |
| RPS27 | AD | Diamond-Blackfan anemia |
| RPS27A | AD | Diamond-Blackfan anemia |
| RPS28 | AD | Diamond-Blackfan anemia |
| RPS29 | AD | Diamond-Blackfan anemia |
| RPS7 | AD | Diamond-Blackfan anemia |
| TSR2 | XLR | Diamond-Blackfan anemia |
| ACD | AD, AR | Dyskeratosis congenita |
| DKC1 | XLR | Dyskeratosis congenita |
| NAF1 | AD | Dyskeratosis congenita |
| NHP2 | AR | Dyskeratosis congenita |
| NOP10 | AR | Dyskeratosis congenita |
| PARN | AD, AR | Dyskeratosis congenita |
| RTEL1 | AD, AR | Dyskeratosis congenita |
| TERC | AD | Dyskeratosis congenita |
| TERT | AD, AR | Dyskeratosis congenita |
| TINF2 | AD | Dyskeratosis congenita |
| WRAP53 | AR | Dyskeratosis congenita |
| CEBPA | AD | Familial AML |
| POT1 | AD | Familial chronic lymphocytic leukemia |
| PRF1 | AR | Familial hemophagocytic lymphohistiocytosis |
| DDX41 | AD | Familial myeloproliferative/lymphoproliferative neoplasms |
| RUNX1 | AD | Familial platelet disorder with associated myeloid malignancy |
| MPL | AD | Familial thrombocythemia |
| BRCA1 | AR | Fanconi anemia |
| BRCA2 | AR | Fanconi anemia |
| BRIP1 | AR | Fanconi anemia |
| ERCC4 | AR | Fanconi anemia |
| FANCA | AR | Fanconi anemia |
| FANCB | XLR | Fanconi anemia |
| FANCC | AR | Fanconi anemia |
| FANCD2 | AR | Fanconi anemia |
| FANCE | AR | Fanconi anemia |
| FANCF | AR | Fanconi anemia |
| FANCG | AR | Fanconi anemia |
| FANCI | AR | Fanconi anemia |
| FANCL | AR | Fanconi anemia |
| FANCM | AR | Fanconi anemia |
| MAD2L2 | AR | Fanconi anemia |
| PALB2 | AR | Fanconi anemia |
| RAD51 | AD | Fanconi anemia |
| RAD51C | AR | Fanconi anemia |
| SLX4 | AR | Fanconi anemia |
| UBE2T | AR | Fanconi anemia |
| XRCC2 | AR | Fanconi anemia |
| FLNA | AD | FNLA-related thrombocytopenia |
| GATA2 | AD | GATA2 deficiency |
| NBEAL2 | AR | Gray platelet syndrome |
| RAB27A | AR | Griscelli syndrome |
| STAT3 | AD | Hyper-IgE recurrent infection syndrome |
| MYD88 | AR | Immunodeficiency |
| TET2 | AR | Immunodeficiency |
| CD40LG | XLR | Immunodeficiency with hyper-IgM, X-linked |
| UNG | AR | Immunodeficiency with hyper IgM |
| AICDA | AR | Immunodeficiency with hyper-IgM |
| CD40 | AR | Immunodeficiency with hyper-IgM |
| TP53 | AD | Li-Fraumeni syndrome |
| LIG4 | AR | LIG4 syndrome |
| CD27 | AR | Lymphoproliferative syndrome |
| CD70 | AR | Lymphoproliferative syndrome |
| ITK | AR | Lymphoproliferative syndrome |
| SH2D1A | XLR | Lymphoproliferative syndrome, X-linked |
| XIAP | XLR | Lymphoproliferative syndrome, X-linked |
| TUBB1 | AD | Macrothrombocytopenia |
| MYH9 | AD | Macrothrombocytopenia and granulocyte inclusions with or without nephritis or sensorineural hearing loss |
| KIT | AD | Mastocytosis |
| MLH1 | AR | Mismatch repair cancer syndrome |
| MSH2 | AR | Mismatch repair cancer syndrome |
| MSH6 | AR | Mismatch repair cancer syndrome |
| PMS2 | AR | Mismatch repair cancer syndrome |
| SAMD9 | AD | Monosomy 7 myelodysplasia and leukemia syndrome, MIRAGE syndrome |
| MPO | AR | Myeloperoxidase deficiency |
| NF1 | AD | Neurofibromatosis |
| NBN | AR | Nijmegen breakage |
| LZTR1 | AD, AR | Noonan syndrome |
| NRAS | AD | Noonan syndrome |
| PTPN11 | AD | Noonan syndrome |
| RAF1 | AD | Noonan syndrome |
| RASA2 | AD | Noonan syndrome |
| RIT1 | AD | Noonan syndrome |
| RRAS | AD | Noonan syndrome |
| SOS1 | AD | Noonan syndrome |
| SOS2 | AD | Noonan syndrome |
| BRAF | AD | Noonan syndrome, Cardiofaciocutaneous syndrome |
| KRAS | AD | Noonan syndrome, Cardiofaciocutaneous syndrome, Autoimmune lymphoproliferative syndrome, type IV / RAS-associated autoimmune leukoproliferative disorder |
| CBL | AD | Noonan syndrome-like disorder |
| SHOC2 | AD | Noonan syndrome-like disorder |
| CA2 | AR | Osteopetrosis |
| CLCN7 | AD, AR | Osteopetrosis |
| LRP5 | AD | Osteopetrosis |
| OSTM1 | AR | Osteopetrosis |
| PLEKHM1 | AD, AR | Osteopetrosis |
| SNX10 | AR | Osteopetrosis |
| TCIRG1 | AR | Osteopetrosis |
| TNFRSF11A | AR | Osteopetrosis |
| TNFSF11 | AR | Osteopetrosis |
| USB1 | AR | Poikiloderma with neutropenia |
| HOXA11 | AD | Radioulnar synostosis with amegakaryocytic thrombocytopenia |
| MECOM | AD | Radioulnar synostosis with amegakaryocytic thrombocytopenia |
| RNF168 | AR | RIDDLE syndrome |
| CSF3R | AR | Severe congenital neutropenia |
| ELANE | AD | Severe congenital neutropenia |
| G6PC3 | AR | Severe congenital neutropenia |
| GFI1 | AD | Severe congenital neutropenia |
| HAX1 | AR | Severe congenital neutropenia |
| JAGN1 | AR | Severe congenital neutropenia |
| VPS45 | AR | Severe congenital neutropenia |
| SRP54 | AD | Severe congenital neutropenia, Shwachman-Diamond-like syndrome |
| ADA | AR | Severe combined immunodeficiency due to ADA deficiency |
| NHEJ1 | AR | Severe combined immunodeficiency with microcephaly, growth retardation, and sensitivity to ionizing radiation |
| DCLRE1C | AR | Severe combined immunodeficiency, Athabascan type |
| RAG1 | AR | Severe combined immunodeficiency, B cell-negative |
| RAG2 | AR | Severe combined immunodeficiency, B cell-negative |
| IL7R | AR | Severe combined immunodeficiency, T cell-negative, B-cell/natural killer-cell positive |
| PTPRC | AR | Severe combined immunodeficiency, T cell-negative, B-cell/natural killer-cell positive |
| JAK3 | AR | Severe combined immunodeficiency, T-negative/B-positive type |
| IL2RG | XLR | Severe combined immunodeficiency, X-linked |
| EFL1 (EFTUD1) | AR | Shwachman-Diamond syndrome |
| SBDS | AR | Shwachman-Diamond syndrome |
| PAX5 | AD | Susceptibility to ALL |
| JAK2 | AD | Thrombocythemia |
| THPO | AD | Thrombocythemia |
| ANKRD26 | AD | Thrombocytopenia |
| CYCS | AD | Thrombocytopenia |
| ETV6 | AD | Thrombocytopenia |
| IKZF5 | AD | Thrombocytopenia |
| PTPRJ | AR | Thrombocytopenia |
| SBF2 | AR | Thrombocytopenia, Charcot-Marie-Tooth disease |
| RBM8A | AR | Thrombocytopenia-absent radius syndrome |
| ADA2 (CECR1) | AR | Vasculitis, autoinflammation, immunodeficiency, and hematologic defects syndrome |
| EZH2 | AD | Weaver syndrome |
| CXCR4 | AD | WHIM syndrome |
| WIPF1 | AR | Wiskott-Aldrich syndrome |
| WAS | XLR | Wiskott-Aldrich syndrome, severe congenital neutropenia, trombocytopenia |

## Supplemental Table 2B. Oncology Panel

| **Gene** | **Inheritance** | **Cancer syndrome, cancer risk** |
| --- | --- | --- |
| AIP | AD | Pituitary adenoma |
| ALK | AD | Neuroblastoma |
| APC | AD | Familial adenomatous polyposis/colorectal cancer, other gastrointestinal cancers, CNS cancer |
| AR | AD | Prostate cancer, male breast cancer |
| ATM | AD | Breast cancer |
| ATR | AD | Oropharyngeal cancer, cutaneous telangiectasia and cancer syndrome, familial |
| AXIN2 | AD | Oligodontia-colorectal cancer syndrome |
| BAP1 | AD | Tumor predisposition syndrome (melanoma, malignant mesothelioma, lung adenocarcinoma, meningioma, and renal cell carcinoma) |
| BARD1 | AD | Breast cancer |
| BLM | AR | Bloom syndrome (squamous cell skin cancer, leukemia, lymphoma, and gastrointestinal tract cancer) |
| BMPR1A | AD | Juvenile polyposis syndrome (stomach, small intestine, colon, and rectum cancer) |
| BRAF | AD | LEOPARD syndrome, Noonan syndrome, Cardiofaciocutaneous syndrome |
| BRCA1 | AD | Breast cancer, ovarian cancer, pancreatic cancer |
| BRCA2 | AD | Breast cancer, ovarian cancer, pancreatic cancer, medulloblastoma, prostate cancer, Wilms tumor |
| BRIP1 | AD | Breast cancer |
| BUB1B | AR | Mosaic variegated aneuploidy syndrome |
| CBL | AD | Noonan syndrome-like disorder |
| CDC73 | AD | Parathyroid carcinoma, hyperparathyroidism-jaw tumor syndrome |
| CDH1 | AD | Gastric cancer, breast cancer, prostate cancer |
| CDK4 | AD | Malignant melanoma, familial |
| CDKN1B | AD | Multiple endocrine neoplasia |
| CDKN1C | AD | Beckwith-Wiedemann syndrome |
| CDKN2A | AD | Pancreatic cancer, familial malignant melanoma |
| CEP57 | AR | Mosaic variegated aneuploidy syndrome |
| CHEK2 | AD | Breast cancer, colorectal cancer |
| CYLD | AD | Brooke-Spiegler syndrome, familial cylindromatosis, multiple familial trichoepithelioma |
| DDB2 | AR | Xeroderma pigmentosum |
| DICER1 | AD | DICER1 syndrome (pleuropulmonary blastoma, Sertoli-Leydig cell tumor, tyhroid cancer) |
| DIS3L2 | AR | Perlman syndrome |
| EGFR | AD | Familial lung cancer |
| EPCAM | AD | Colorectal cancer, endometrial cancer |
| ERCC2 | AR | Xeroderma pigmentosum |
| ERCC3 | AR | Xeroderma pigmentosum |
| ERCC4 | AR | Xeroderma pigmentosum |
| ERCC5 | AR | Xeroderma pigmentosum |
| EXT1 | AD | Multiple exostoses |
| EXT2 | AD | Multiple exostoses |
| FAM111B | AD | Pancreatic cancer (POIKTMP - Poikiloderma, hereditary fibrosing, with tendon contractures, myopathy, and pulmonary fibrosis) |
| FANCM | AD | Breast cancer |
| FH | AD | Hereditary leiomyomatosis and renal cell cancer |
| FLCN | AD | Birt-Hogg-Dube syndrome |
| GALNT12 | AD | Colorectal cancer |
| GPC3 | XLR | Simpson Golabi Behmel syndrome |
| GREM1 | AD | Hereditary Mixed Polyposis Syndrome |
| HNF1A | AR | Hepatocellular carcinoma, hepatic adenoma, renal cell carcinoma |
| HOXB13 | AD | Familial prostate cancer |
| HRAS | AD | Costello syndrome |
| KIT | AD | Gastrointestinal stromal tumor |
| KRAS | AD | Noonan syndrome, Cardiofaciocutaneous syndrome |
| LZTR1 | AD, AR | Noonan syndrome, schwannomatosis |
| MAP2K1 | AD | Cardiofaciocutaneous syndrome |
| MAP2K2 | AD | Cardiofaciocutaneous syndrome |
| MAX | AD | Pheochromocytoma |
| MEN1 | AD | Multiple endocrine neoplasia |
| MET | AD | Hereditary papillary renal carcinoma |
| MITF | AD | Renal cancer, melanoma |
| MLH1 | AD; AR | Muir-Torre syndrome, hereditary nonpolyposis colorectal cancer; Mismatch repair cancer syndrome |
| MLH3 | AD | Hereditary nonpolyposis colorectal cancer, endometrial cancer |
| MSH2 | AD; AR | Muir-Torre syndrome, hereditary nonpolyposis colorectal cancer; Mismatch repair cancer syndrome |
| MSH3 | AR | Familial adenomatous polyposis |
| MSH6 | AD; AR | Hereditary nonpolyposis colorectal cancer, endometrial cancer; Mismatch repair cancer syndrome |
| MUTYH | AR | Familial adenomatous polyposis (MUTYH-associated polyposis syndrome) |
| NBN | AR | Nijmegen breakage syndrome |
| NF1 | AD | Neurofibromatosis |
| NF2 | AD | Neurofibromatosis |
| NRAS | AD | Noonan syndrome |
| NTHL1 | AR | Familial adenomatous polyposis |
| PALB2 | AD | Breast cancer, pancreatic cancer, gastrointestinal cancer |
| PDGFRA | AD | Familial gastrointestinal stromal tumour |
| PHOX2B | AD | Neuroblastoma |
| PMS2 | AD; AR | Hereditary nonpolyposis colorectal cancer; Mismatch repair cancer syndrome |
| POLD1 | AD | Colorectal cancer |
| POLE | AD | Colorectal cancer |
| POLH | AR | Xeroderma pigmentosum |
| POT1 | AD | Malignant melanoma, glioma |
| PRKAR1A | AD | Carney complex |
| PTCH1 | AD | Basal cell nevus syndrome |
| PTEN | AD | Cowden syndrome |
| PTPN11 | AD | Noonan syndrome |
| RAD51C | AD | Breast-ovarian cancer |
| RAD51D | AD | Breast-ovarian cancer |
| RAF1 | AD | Noonan syndrome |
| RASA2 | AD | Noonan syndrome |
| RB1 | AD | Retinoblastoma |
| RECQL4 | AR | Rothmund-Thomson syndrome |
| REST | AD | Wilms tumor |
| RET | AD | Multiple endocrine neoplasia |
| RHBDF2 | AD | Tylosis with esophageal cancer |
| RIT1 | AD | Noonan syndrome |
| RPS20 | AD | Colorectal cancer |
| RRAS | AD | Noonan-like syndrome |
| SDHA | AD | Paraganglioma, pheochromocytoma |
| SDHAF2 | AD | Paraganglioma, pheochromocytoma |
| SDHB | AD | Paraganglioma, pheochromocytoma, gastrointestinal stromal tumor |
| SDHC | AD | Paraganglioma, pheochromocytoma, gastrointestinal stromal tumor |
| SDHD | AD | Paraganglioma, pheochromocytoma, gastrointestinal stromal tumor |
| SHOC2 | AD | Noonan-like syndrome |
| SMAD4 | AD | Juvenile polyposis/hereditary hemorrhagic telangiectasia syndrome |
| SMARCA4 | AD | Rhabdoid tumor predisposition syndrome |
| SMARCB1 | AD | Rhabdoid tumor predisposition syndrome, schwannomatosis |
| SOS1 | AD | Noonan syndrome |
| SOS2 | AD | Noonan syndrome |
| STK11 | AD | Peutz-Jeghers syndrome |
| SUFU | AD | Basal cell nevus syndrome, medulloblastoma |
| TERT | AD | Malignant melanoma |
| TMEM127 | AD | Pheochromocytoma, paraganglioma |
| TP53 | AD | Li-Fraumeni syndrome |
| TSC1 | AD | Tuberous sclerosis |
| TSC2 | AD | Tuberous sclerosis |
| VHL | AD | Von Hippel-Lindau syndrome |
| WRN | AR | Werner syndrome |
| WT1 | AD | Wilms tumor |
| XPA | AR | Xeroderma pigmentosum |
| XPC | AR | Xeroderma pigmentosum |

## Supplemental Table 2C. ACMG SF v3.0 Panel

| **Gene** | **MIM number** | **Disease (MIM number)** |
| --- | --- | --- |
| APC | 611731 | Adenomatous polyposis coli (MIM 175100) |
| MYH11 | 160745 | Aortic aneurysm, familial thoracic 4 (MIM 132900) |
| ACTA2 | 102620 | Aortic aneurysm, familial thoracic 6 (MIM 611788) |
| TMEM43 | 612048 | Arrhythmogenic right ventricular cardiomyopathy, type 5 (MIM 604400) |
| DSP | 125647 | Arrhythmogenic right ventricular cardiomyopathy, type 8 (MIM 607450) |
| PKP2 | 602861 | Arrhythmogenic right ventricular cardiomyopathy, type 9 (MIM 609040) |
| DSG2 | 125671 | Arrhythmogenic right ventricular cardiomyopathy, type 10 (MIM 610193) |
| DSC2 | 125645 | Arrhythmogenic right ventricular cardiomyopathy, type 11 (MIM 610476) |
| BTD | 609019 | Biotinidase deficiency (MIM 253260) |
| BRCA1 | 113705 | Breast-ovarian cancer, familial 1 (MIM 604370) |
| BRCA2 | 600185 | Breast-ovarian cancer, familial 2 (MIM 612555) |
| SCN5A | 600163 | Brugada syndrome 1 (MIM 601144) |
| CASQ2 | 114251 | Catecholaminergic polymorphic ventricular tachycardia (MIM 604772) |
| RYR2 | 180902 | Catecholaminergic polymorphic ventricular tachycardia (MIM 604772) |
| FLNC | 102565 | Dilated cardiomyopathy (MIM 617047) |
| TTN | 188840 | Dilated cardiomyopathy (truncating variants only) (MIM 604145) |
| LMNA | 150330 | Dilated cardiomyopathy 1A (MIM 115200) |
| MYBPC3 | 600958 | Dilated cardiomyopathy 1A (MIM 115200) |
| COL3A1 | 120180 | Ehlers-Danlos syndrome, type 4 (MIM 130050) |
| GLA | 300644 | Fabry's disease (MIM 301500) |
| APOB | 107730 | Familial hypercholesterolemia (MIM 143890) |
| LDLR | 606945 | Familial hypercholesterolemia (MIM 143890) |
| MYH7 | 160760 | Familial hypertrophic cardiomyopathy 1 (MIM 192600) |
| TPM1 | 191010 | Familial hypertrophic cardiomyopathy 3 (MIM 115196) |
| MYBPC3 | 600958 | Familial hypertrophic cardiomyopathy 4 (MIM 115197) |
| PRKAG2 | 602743 | Familial hypertrophic cardiomyopathy 6 (MIM 600858) |
| TNNI3 | 191044 | Familial hypertrophic cardiomyopathy 7 (MIM 613690) |
| MYL3 | 160790 | Familial hypertrophic cardiomyopathy 8 (MIM 608751) |
| MYL2 | 160781 | Familial hypertrophic cardiomyopathy 10 (MIM 608758) |
| ACTC1 | 102540 | Familial hypertrophic cardiomyopathy 11 (MIM 612098) |
| RET | 164761 | Familial medullary thyroid carcinoma (MIM 155240) |
| PALB2 | 610355 | Hereditary breast cancer (MIM 114480) |
| HFE | 613609 | Hereditary hemochromatosis (c.845G>A; p.C282Y homozygotes only) (MIM 235200) |
| ENG | 131195 | Hereditary hemorrhagic telangiectasia type 1 (MIM 187300) |
| ACVRL1 | 601284 | Hereditary hemorrhagic telangiectasia type 2 (MIM 600376) |
| MAX | 154950 | Hereditary paraganglioma-pheochromocytoma syndrome (MIM 171300) |
| TMEM127 | 613403 | Hereditary paraganglioma-pheochromocytoma syndrome (MIM 171300) |
| PCSK9 | 607786 | Hypercholesterolemia, autosomal dominant, 3 (MIM 603776) |
| BMPR1A | 601299 | Juvenile polyposis syndrome (MIM 174900) |
| SMAD4 | 600993 | Juvenile polyposis syndrome (MIM 174900) |
| TNNT2 | 191045 | Left ventricular noncompaction 6 (MIM 601494) |
| TP53 | 191170 | Li-Fraumeni syndrome 1 (MIM 151623) |
| TGFBR1 | 190181 | Loeys-Dietz syndrome type 1A (MIM 609192) |
| TGFBR2 | 190182 | Loeys-Dietz syndrome type 1B (MIM 610168) |
| TGFBR1 | 190181 | Loeys-Dietz syndrome type 2A (MIM 608967) |
| TGFBR2 | 190182 | Loeys-Dietz syndrome type 2B (MIM 610380) |
| SMAD3 | 603109 | Loeys-Dietz syndrome type 3 (MIM 613795) |
| TRDN | 607542 | Long QT syndrome |
| KCNQ1 | 607542 | Long QT syndrome 1 (MIM 192500) |
| KCNH2 | 152427 | Long QT syndrome 2 (MIM 613688) |
| SCN5A | 600163 | Long QT syndrome 3 (MIM 603830) |
| MLH1 | 120436 | Lynch syndrome (MIM 120435) |
| MSH2 | 609309 | Lynch syndrome (MIM 120435) |
| MSH6 | 600678 | Lynch syndrome (MIM 120435) |
| PMS2 | 600259 | Lynch syndrome (MIM 120435) |
| RYR1 | 180901 | Malignant hyperthermia (MIM 145600) |
| CACNA1S | 114208 | Malignant hyperthermia (MIM 145600) |
| FBN1 | 134797 | Marfan's syndrome (MIM 154700) |
| TGFBR1 | 190181 | Marfan's syndrome (MIM 154700) |
| HNF1A | 142410 | Maturity-Onset of Diabetes of the Young (MIM 600496) |
| MEN1 | 613733 | Multiple endocrine neoplasia, type 1 (MIM 131100) |
| RET | 164761 | Multiple endocrine neoplasia, type 2a (MIM 171400) |
|  |  | Multiple endocrine neoplasia, type 2b (MIM 162300) |
| MUTYH | 604933 | MYH-associated polyposis (MIM 608456) |
| NF2 | 607379 | Neurofibromatosis, type 2 (MIM 101000) |
| OTC | 300461 | Ornithine carbamoyltransferase deficiency (MIM 311250) |
| SDHD | 602690 | Paragangliomas 1 (MIM 168000) |
| SDHAF2 | 613019 | Paragangliomas 2 (MIM 601650) |
| SDHC | 602413 | Paragangliomas 3 (MIM 605373) |
| SDHB | 185470 | Paragangliomas 4 (MIM 115310) |
| STK11 | 602216 | Peutz-Jeghers syndrome (MIM 175200) |
| MUTYH | 604933 | Pilomatrixoma (MIM 132600) |
| GAA | 604933 | Pompe disease (MIM 232300) |
| PTEN | 601728 | PTEN hamartoma tumor syndrome (MIM 153480) |
| RB1 | 614041 | Retinoblastoma (MIM 180200) |
| RPE65 | 180069 | RPE65-related retinopathy (MIM 204100, MIM 613794) |
| TSC1 | 605284 | Tuberous sclerosis 1 (MIM 191100) |
| TSC2 | 191092 | Tuberous sclerosis 2 (MIM 613254) |
| VHL | 608537 | Von Hippel-Lindau syndrome (MIM 193300) |
| WT1 | 607102 | Wilms' tumor (MIM 194070) |
| ATP7B | 606882 | Wilson disease (MIM 277900) |

Supplemental Table 3. Harmful variants in analyzed genes. A) Adult cohort 1, B) Adult cohort 2, C) Pediatric cohort. *Patient carries more than one variant in analyzed genes. **Causative mutation identified before hematopoietic stem cell transplantation. X included in gene panel, (X) included in gene panel with different genotype (not included in results), - Not included in gene panel. AA - Aplastic anemia, AF - Allele frequency, ALD - Adrenoleukodystrophy, ALL - Acute lymphocytic leukemia, Alt - Alternative sequence, AML - Acute myeloid leukemia, BMF - Bone marrow failure, CGD - Chronic granulomatous disease, CIP - Conflicting interpretation of pathogenicity, CLL - Chronic lymphocytic leukemia, CML - Chronic myeloid leukemia, CMML - Chronic myelomonocytic leukemia, Het - Heterozygous, HIGM - Hyper IgM syndrome, Hom - Homozygous, HSCT - Hematopoietic stem cell transplantation LP - Likely pathogenic, M - Moderate, MDS - Myelodysplastic syndrome, MM - Multiple myeloma, MPN - Myeloproliferative neoplasm, OPT - Osteopetrosis, P - Pathogenic, PCL - Plasma cell leukemia, PID - Primary immunodeficiency, Ref - Reference sequence, SCID - Severe combined immunodeficiency, St - Strong, Su - Supporting, UNK - Unknown, VAF - Variant allele frequency, VS - Very Strong, VUS - Variant of uncertain significance, WAS - Wiskott-Aldrich syndrome, XLP - X-linked lymphoproliferative disease.

## Supplemental Table 3A. Adult cohort 1

| **Patient** | **Diagnosis** | **Age at HSCT** | **Family Donor** | **Gene** | **Amino acid change (Transcript)** | **Genotype** | **VAF** | **Clinvar** | **InterVar (Criteria)** | **Varsome (Criteria)** | **Hematology Panel** | **Oncology Panel** | **ACMG SF v3.0 Panel** | **AF (Fin)** | **AF (All)** |
| --- | --- | --- | --- | --- | --- | --- | --- | --- | --- | --- | --- | --- | --- | --- | --- |
| D028* | AML** | 33 | Yes | CEBPA | p.Gln311Pro (1) (NM_004364.5:c.932A>C) | Het(66/127) | 0.52 | UNK | VUS (PM1, PM2, PP3, BP1) | VUS (PM1 M, PM2 M, PP3 Su) | X | - | - | 0 | 0 |
| D084 | AML | 64 | No | DDX41 | p.Met1Ile  (NM_016222.4:c.3G>A) | Het(18/45) | 0.40 | P / LP | VUS (PP3, PP5) | P (PVS1 VS, PP5 St, PP3 Su) | X | - | - | 0.00005 | 0.00009 |
| D063 | BMF** | 18 | No | ERCC6L2 | p.Ile475ThrfsTer36 (2) (NM_020207.7:c.1424del) | Hom(45/45) | 1.00 | UNK | VUS (PVS1) | P (PVS1 VS, PP5 St, PM2 M, PP3 Su) | X | - | - | 0.00511 | 0.00048 |
| D049 | AML | 38 | No | ERCC6L2 | p.Ile475ThrfsTer36 (2) (NM_020207.7:c.1424del) | Hom(97/97) | 1.00 | UNK | VUS (PVS1) | P (PVS1 VS, PP5 St, PM2 M, PP3 Su) | X | - | - | 0.00511 | 0.00048 |
| D021 | MDS** | 41 | No | GATA2 | p.Thr354Met (NM_032638.5:c.1061C>T) | Het(43/99) | 0.43 | P | LP (PM1, PM2, PP3, PP5) | P (PP5 VS, PM1 M, PM2 M, PM5 M, PP2 Su, PP3 Su) | X | - | - | 0 | 0 |
| D029 | MDS** | 32 | No | GATA2 | p.Gln328Ter  (NM_032638.5:c.982C>T) | Het(38/79) | 0.48 | UNK | P (PVS1, PM2, PP3) | P (PVS1 VS, PM2 M, PP3 Su) | X | - | - | 0 | 0 |
| D120* | MDS** | 19 | No | GATA2 | p.Thr354Met (NM_032638.5:c.1061C>T) | Het(54/122) | 0.44 | P | LP (PM1, PM2, PP3, PP5) | P (PP5 VS, PM1 M, PM2 M, PM5 M, PP2 Su, PP3 Su) | X | - | - | 0 | 0 |
| D099 | MM | 56 | No | POT1 | Splicing  (NM_015450.3:c.547-1G>A) | Het(31/51) | 0.61 | UNK | P (PVS1, PM2, PP3) | P (PVS1 VS, PM2 M, PP3 Su) | X | X | - | 0 | 0 |
| D011 | ALL | 55 | Yes | RUNX1 | p.Arg204Gln (NM_001754.5:c.611G>A) | Het(32/70) | 0.46 | P | LP (PM1, PM2, PP3, PP5) | P (PP5 VS, PP3 St, PM1 M, PM2 M, PM5 M, PP2 Su) | X | - | - | 0 | 0 |
| D141 | MDS/MPN | 59 | No | TERT | p.Arg774Ter (NM_198253.3:c.2320C>T) | Het(59/114) | 0.52 | P | P (PVS1, PM2, PP5) | P (PVS1 VS, PM2 M, PP5 M, BP4 Su) | X | X | - | 0.00052 | 0.00006 |
| D027 | AML | 46 | No | AR | p.Arg727Leu (NM_000044.6:c.2180G>T) | Hom(48/48) | 1.00 | P | VUS (PM1, PP3, PP5) | LP (PM1 M, PP2 Su, PP3 Su, PP5 Su, BS2 St) | - | X | - | 0.00835 | 0.00095 |
| D042 | AML | 57 | No | ATR | p.Glu2155Ter (NM_001184.4:c.6463G>T) | Het(50/117) | 0.43 | UNK | P (PVS1, PM2, PP3) | P (PVS1 VS, PM2 M, PP3 Su) | - | X | - | 0 | 0 |
| D004 | ALL | 41 | No | BRCA1 | Splicing  (NM_007300.4:c.4097-2A>G) | Het(14/24) | 0.58 | P | P (PVS1, PM2, PP3, PP5) | P (PVS1 VS, PP5 VS, PM2 M, PP3 Su) | (X) | X | X | 0 | 0 |
| D111 | AML | 65 | No | BRCA2 | p.Arg3128Ter (NM_000059.4:c.9382C>T) | Het(38/74) | 0.51 | P | P (PVS1, PM2, PP3, PP5) | P (PVS1 VS, PP5 VS, PM2 M, PP3 Su) | (X) | X | X | 0 | 0.00001 |
| D104 | AML | 59 | Yes | CHEK2 | p.Thr410MetfsTer15 (3) (NM_001005735.2:c.1229del) | Het(8/16) | 0.50 | CIP | VUS (PVS1) | P (PVS1 VS, PP5 St, PM2 M, PP3 Su) | - | X | - | 0.00874 | 0.00205 |
| D013 | AML | 70 | No | CHEK2 | p.Thr410MetfsTer15 (3) (NM_001005735.2:c.1229del) | Het(20/30) | 0.67 | CIP | VUS (PVS1) | P (PVS1 VS, PP5 St, PM2 M, PP3 Su) | - | X | - | 0.00874 | 0.00205 |
| D074 | AML | 45 | No | CHEK2 | p.Thr410MetfsTer15 (3) (NM_001005735.2:c.1229del) | Het(16/29) | 0.55 | CIP | VUS (PVS1) | P (PVS1 VS, PP5 St, PM2 M, PP3 Su) | - | X | - | 0.00874 | 0.00205 |
| D089 | ALL | 33 | No | CHEK2 | p.Thr410MetfsTer15 (3) (NM_001005735.2:c.1229del) | Het(24/56) | 0.43 | CIP | VUS (PVS1) | P (PVS1 VS, PP5 St, PM2 M, PP3 Su) | - | X | - | 0.00874 | 0.00205 |
| D136 | AA | 17 | No | EGFR | p.Pro848Leu (NM_005228.5:c.2543C>T) | Het(21/38) | 0.55 | CIP | LP (PM1, PM2, PP2, PP3) | P (PP5 VS, PM1 M, PM2 M, PP2 Su, PP3 Su, BS3 St) | - | X | - | 0.00023 | 0.00036 |
| D086 | CMML | 66 | No | FANCM | p.Gln1701Ter (4) (NM_020937.4:c.5101C>T) | Het(60/129) | 0.47 | P / LP | VUS (PVS1, PP5) | P (PVS1 VS, PP5 VS, PM2 M, BP4 Su) | (X) | X | - | 0.00823 | 0.00129 |
| D010 | AML | 47 | Yes | FANCM | p.Gln498ThrfsTer7 (5) (NM_020937.4:c.1491dup) | Het(75/149) | 0.50 | P / LP | VUS (PVS1, PP5) | P (PVS1 VS, PP5 VS, PM2 M, PP3 Su) | (X) | X | - | 0.00023 | 0.00004 |
| D103 | AML | 45 | No | FANCM | p.Arg1931Ter (6) (NM_020937.4:c.5791C>T) | Het(104/248) | 0.42 | P | P (PVS1, PP3, PP5) | P (PVS1 VS, PM2 M, PP3 Su) | (X) | X | - | 0.00448 | 0.00101 |
| D062 | AML | 37 | Yes | HOXB13 | p.Gly84Glu (7) (NM_006361.6:c.251G>A) | Het(30/42) | 0.71 | P / LP | VUS (PM1, PP2, PP3, PP5) | VUS (PP5 St, PP3 Su) | - | X | - | 0.00783 | 0.00186 |
| D088 | ALL | 48 | No | HOXB13 | p.Gly84Glu (7) (NM_006361.6:c.251G>A) | Het(33/60) | 0.55 | P / LP | VUS (PM1, PP2, PP3, PP5) | VUS (PP5 St, PP3 Su) | - | X | - | 0.00783 | 0.00186 |
| D120* | MDS | 19 | No | HOXB13 | p.Gly84Glu (7) (NM_006361.6:c.251G>A) | Het(233/504) | 0.46 | P / LP | VUS (PM1, PP2, PP3, PP5) | VUS (PP5 St, PP3 Su) | - | X | - | 0.00783 | 0.00186 |
| D028* | AML | 33 | Yes | PALB2 | p.Asp219ThrfsTer4 (NM_024675.4:c.654del) | Het(101/192) | 0.53 | P | P (PVS1, PM2, PP5) | P (PVS1 VS, PP5 St, PM2 M) | (X) | X | X | 0 | 0.000004 |

## Supplemental Table 3B. Adult cohort 2

| **Patient** | **Diagnosis** | **Age at HSCT** | **Family Donor** | **Gene** | **Amino acid change (Transcript)** | **Genotype** | **VAF** | **Clinvar** | **InterVar (Criteria)** | **Varsome (Criteria)** | **Hematology Panel** | **Oncology Panel** | **ACMG SF v3.0 Panel** | **AF (Fin)** | **AF (All)** |
| --- | --- | --- | --- | --- | --- | --- | --- | --- | --- | --- | --- | --- | --- | --- | --- |
| V062 | AA | 43 | Yes | ADA2 | p.Arg169Gln (NM_001282225.2:c.506G>A) | Hom(12/12) | 1.00 | P / LP | VUS (PM2, PP3, PP5) | P (PP5 VS, PM1 M, PM2 M, PM5- M, PP2 Su, PP3 Su) | X | - | - | 0.0019 | 0.00048 |
| V075 | AML | 36 | Yes | ANKRD26 | p.Glu696GlyfsTer3 (NM_014915.3:c.2087_2088del) | Het(10/22) | 0.45 | UNK | LP (PVS1, PM2) | P (PVS1 VS, PM2 M, PP3 Su) | X | - | - | 0 | 0 |
| V038 | ALL | 48 | Yes | ANKRD26 | p.Glu1545Ter (NM_014915.3:c.4633G>T) | Het(24/40) | 0.60 | UNK | LP (PVS1, PM2) | P (PVS1 VS, PM2 M, PP3 Su) | X | - | - | 0 | 0.000004 |
| V134 | AML | 44 | Yes | GATA2 | p.Thr354Met (NM_032638.5:c.1061C>T) | Het(5/15) | 0.33 | P | LP (PM1, PM2, PP3, PP5) | P (PP5 VS, PM1 M, PM2 M, PM5 M, PP2 Su, PP3 Su) | X | - | - | 0 | 0 |
| V054 | AML | 63 | Yes | SAMD9 | p.Arg344Ter (NM_001193307.1:c.1030C>T) | Het(19/38) | 0.50 | P | VUS (PM2, PP5) | P (PVS1 VS, PM2 M, PP5 Su, BP4 Su) | X | - | - | 0 | 0.00004 |
| V073 | CLL | 61 | Yes | ATM | Splicing (NM_000051.4:c.6198+1G>A) | Het(14/28) | 0.50 | P / LP | P (PVS1, PM2, PP3, PP5) | P (PVS1 VS, PP5 St, PM2 M, PP3 Su) | (X) | X | - | 0 | 0.000004 |
| V136 | AML | 60 | Yes | CHEK2 | p.Thr410MetfsTer15 (3) (NM_001005735.2:c.1229del) | Het(4/12) | 0.33 | CIP | VUS (PVS1) | P (PVS1 VS, PP5 St, PM2 M, PP3 Su) | - | X | - | 0.00874 | 0.00205 |
| V052 | ALL | 18 | Yes | CHEK2 | p.Thr410MetfsTer15 (3) (NM_001005735.2:c.1229del) | Het(5/10) | 0.50 | CIP | VUS (PVS1) | P (PVS1 VS, PP5 St, PM2 M, PP3 Su) | - | X | - | 0.00874 | 0.00205 |
| V122 | AML | 41 | Yes | CHEK2 | p.Thr410MetfsTer15 (3) (NM_001005735.2:c.1229del) | Het(10/12) | 0.83 | CIP | VUS (PVS1) | P (PVS1 VS, PP5 St, PM2 M, PP3 Su) | - | X | - | 0.00874 | 0.00205 |
| V117 | CLL | 52 | Yes | CHEK2 | Splicing (NM_001005735.2:c.319+2T>A) | Het(22/40) | 0.55 | P / LP | P (PVS1, PM2, PP3, PP5) | P (PVS1 VS, PP5 St, PM2 M, PP3 Su) | - | X | - | 0.00056 | 0.00007 |
| V012 | AML | 28 | Yes | FANCM | p.Gln1701Ter (4) (NM_020937.4:c.5101C>T) | Het(24/41) | 0.59 | P / LP | VUS (PVS1, PP5) | P (PVS1 VS, PP5 VS, PM2 M, BP4 Su) | (X) | X | - | 0.00823 | 0.00129 |
| V070 | AML | 59 | Yes | FANCM | p.Gln1701Ter (4) (NM_020937.4:c.5101C>T) | Het(24/49) | 0.49 | P / LP | VUS (PVS1, PP5) | P (PVS1 VS, PP5 VS, PM2 M, BP4 Su) | (X) | X | - | 0.00823 | 0.00129 |
| V039 | PCL | 50 | Yes | FANCM | p.Gln1701Ter (4) (NM_020937.4:c.5101C>T) | Het(24/40) | 0.60 | P / LP | VUS (PVS1, PP5) | P (PVS1 VS, PP5 VS, PM2 M, BP4 Su) | (X) | X | - | 0.00823 | 0.00129 |
| V072 | AML | 53 | Yes | HOXB13 | p.Gly84Glu (7) (NM_006361.6:c.251G>A) | Het(5/10) | 0.50 | P / LP | VUS (PM1, PP2, PP3, PP5) | VUS (PP5 St, PP3 Su) | - | X | - | 0.00783 | 0.00186 |
| V021 | MDS | 54 | Yes | RAD51D | Splicing (NM_001142571.2:c.636+1G>A) | Het(7/9) | 0.78 | P / LP | P (PVS1, PP3, PP5) | P (PVS1 VS, PP5 St, PM2 M, PP3 Su) | - | X | - | 0 | 0.000004 |
| V014 | AML | 59 | Yes | WT1 | p.Glu158Ter (NM_024426.6:c.472G>T) | Het(4/10) | 0.40 | P | P (PVS1, PM2, PP3, PP5) | P (PVS1 VS, PM2 M, PP3 Su, PP5 Su) | - | X | X | 0 | 0 |
| V112 | ALL | 46 | Yes | MYH7 | p.Arg1344Trp (NM_000257.4:c.4030C>T) | Het(9/14) | 0.64 | VUS | LP (PM1, PM2, PP2, PP3) | LP (PM2 M, PM5 M, PP2 Su, PP3 Su) | - | - | X | 0 | 0.00002 |
| V006 | AML | 53 | Yes | OTC | p.Arg277Gln (NM_000531.6:c.830G>A) | Het(8/25) | 0.32 | P | LP (PM1, PM2, PP3, PP5) | P (PP5 VS, PM2 M, PM5 M, PP2 Su, PP3 Su) | - | - | X | 0 | 0 |

## Supplemental Table 3C. Pediatric cohort

| **Patient** | **Diagnosis** | **Age at HSCT** | **Family Donor** | **Gene** | **Amino acid change (Transcript)** | **Genotype** | **VAF** | **Clinvar** | **InterVar (Criteria)** | **Varsome (Criteria)** | **Hematology Panel** | **Oncology Panel** | **ACMG SF v3.0 Panel** | **AF (Fin)** | **AF (All)** |
| --- | --- | --- | --- | --- | --- | --- | --- | --- | --- | --- | --- | --- | --- | --- | --- |
| C010* | Other (ALD)** | 5 | No | ABCD1 | p.Arg554His (NM_000033.4:c.1661G>A) | Hom(179/179) | 1.00 | P | LP (PM1, PM2, PP3, PP5) | P (PP5 VS, PM1 M, PM2 M, PM5 M, PP2 Su, PP3 Su) | X | - | - | 0 | 0 |
| C071 | PID (ADA2 deficiency)** | 9 | No | ADA2 | p.Arg169Gln (NM_001282225.2:c.506G>A) | Het(71/179) | 0.40 | P / LP | VUS (PM2, PP3, PP5) | P (PP5 VS, PM1 M, PM2 M, PM5- M, PP2 Su, PP3 Su) | X | - | - | 0.0019 | 0.00048 |
|  |  |  |  | ADA2 | p.Leu311Arg (8) (NM_001282225.2:c.932T>G) | Het(85/151) | 0.56 | UNK | VUS (PM1, PM2, PP3) | LP (PM1 M, PM2 M, PP2 Su, PP3 Su) | X | - | - | 0.00046 | 0.00005 |
| C137 | AA** | 15 | No | ANKRD26 | Splicing  (NM_014915.3:c.1636-2A>T) | Het(34/77) | 0.44 | UNK | P (PVS1, PM2, PP3) | P (PVS1 VS, PM2 M, PP3 Su) | X | - | - | 0.00005 | 0.000005 |
| C113 | PID (HIGM)** | 1 | No | CD40LG | p.Cys218Ter (9) (NM_000074.3:c.654C>A) | Hom(82/82) | 1.00 | UNK | VUS (PM2) | P (PVS1 VS, PM2 M, PP5 M) | X | - | - | 0 | 0 |
| C088 | PID (CGD)** | 14 | Yes | CYBB | Splicing  (NM_000397.4:c.1315-1G>T) | Hom(88/88) | 1.00 | UNK | P (PVS1, PM2, PP3) | P (PVS1 VS, PM2 M, PP3 Su) | X | - | - | 0 | 0 |
| C049 | PID (CGD)** | 3 | Yes | CYBB | Splicing  (NM_000397.4:c.45+1G>T) | Hom(62/62) | 1.00 | UNK | P (PVS1, PM2, PP3) | P (PVS1 VS, PM2 M, PP3 Su) | X | - | - | 0 | 0 |
| C084* | BMF** | 6 | No | FANCG | Splicing  (NM_004629.2:c.1076+1G>A) | Het(99/234) | 0.42 | UNK | P (PVS1, PM2, PP3) | P (PVS1 VS, PM2 M, PP3 Su) | X | - | - | 0.00005 | 0.000004 |
|  |  |  |  | FANCG | p.Glu395TrpfsTer5 (NM_004629.2:c.1183_1192del) | Het(69/124) | 0.56 | P | VUS (PVS1, PP5) | P (PVS1 VS, PM2 M, PP3 Su, PP5 Su) | X | - | - | 0.0001 | 0.00005 |
| C144 | AML** | 12 | No | GATA2 | p.Thr354Met (NM_032638.5:c.1061C>T) | Het(139/279) | 0.50 | P | LP (PM1, PM2, PP3, PP5) | P (PP5 VS, PM1 M, PM2 M, PM5 M, PP2 Su, PP3 Su) | X | - | - | 0 | 0 |
| C122 | PID (SCID) | 0 | No | IL2RG | p.Arg226Cys  (NM_000206.3:c.676C>T) | Hom(111/111) | 1.00 | P | LP (PM1, PM2, PP3, PP5) | P (PP5 VS, PM1 M, PM2 M, PM5 M, PP2 Su, PP3 Su) | X | - | - | 0 | 0 |
| C047 | PID (SCID) | 0 | No | LIG4 | p.Gly276Asp (10) (NM_206937.2:c.827G>A) | Hom(140/140) | 1.00 | UNK | VUS (PM1, PP3) | VUS (PM2 M, PP3 Su) | X | - | - | 0.00056 | 0.00005 |
| C037* | ALL | 3 | Yes | LZTR1 | Splicing  (NM_006767.4:c.2407-1G>A) | Hom(105/111) | 0.95 | UNK | P (PVS1, PM2, PP3) | P (PVS1 VS, PM2 M, PP3 Su) | X | X | - | 0.00018 | 0.00002 |
| C058 | Other (OPT)** | 0 | Yes | TCIRG1 | p.Gln433Ter (NM_006019.4:c.1297C>T) | Hom(382/382) | 1.00 | CIP | LP (PVS1, PM2) | P (PVS1 VS, PM2 M, PP3 Su) | X | - | - | 0.00019 | 0.00005 |
| C092* | AA | 9 | No | TERT | p.Arg774Ter (NM_198253.3:c.2320C>T) | Het(104/222) | 0.47 | P | P (PVS1, PM2, PP5) | P (PVS1 VS, PM2 M, PP5 M, BP4 Su) | X | X | - | 0.00052 | 0.00006 |
| C152* | ALL | 5 | No | TP53 | p.Gly245Ser (ENST00000269305.4:c.733G>A) | Het(77/183) | 0.42 | P | LP (PM1, PM2, PM5, PP3, PP5) | P (PP5 VS, PM1 St, PM2 M, PM5 M, PP2 Su, PP3 Su) | X | X | X | 0 | 0 |
| C025 | ALL | 14 | No | TP53 | p.His179Tyr (ENST00000269305.4:c.535C>T) | Het(215/255) | 0.84 | P / LP | LP (PM1, PM2, PM5, PP3, PP5) | P (PP5 VS, PM1 St, PM2 M, PM5 M, PP2 Su, PP3 Su) | X | X | X | 0 | 0 |
| C140 | BMF (WAS)** | 4 | Yes | WAS | p.Met6AsnfsTer32 (NM_000377.3:c.11dup) | Hom(234/234) | 1.00 | UNK | LP (PVS1, PM2) | P (PVS1 VS, PM2 M, PP3 Su) | X | - | - | 0 | 0 |
| C118 | PID (XLP)** | 5 | No | XIAP | p.Lys297Ter (NM_001204401.2:c.889_892del) | Hom(81/81) | 1.00 | UNK | LP (PVS1, PM2) | P (PVS1 VS, PM2 M, PP3 Su) | X | - | - | 0 | 0 |
| C093 | ALL | 16 | No | AR | p.Arg727Leu (NM_000044.6:c.2180G>T) | Hom(163/163) | 1.00 | P | VUS (PM1, PP3, PP5) | LP (PM1 M, PP2 Su, PP3 Su, PP5 Su, BS2 St) | - | X | - | 0.00835 | 0.00095 |
| C131 | ALL | 9 | No | CHEK2 | p.Thr410MetfsTer15 (3) (NM_001005735.2:c.1229del) | Het(53/110) | 0.48 | CIP | VUS (PVS1) | P (PVS1 VS, PP5 St, PM2 M, PP3 Su) | - | X | - | 0.00874 | 0.00205 |
| C024 | ALL | 16 | No | CHEK2 | p.Thr410MetfsTer15 (3) (NM_001005735.2:c.1229del) | Het(66/133) | 0.50 | CIP | VUS (PVS1) | P (PVS1 VS, PP5 St, PM2 M, PP3 Su) | - | X | - | 0.00874 | 0.00205 |
| C008 | ALL | 16 | No | CHEK2 | p.Thr410MetfsTer15 (3) (NM_001005735.2:c.1229del) | Het(53/113) | 0.47 | CIP | VUS (PVS1) | P (PVS1 VS, PP5 St, PM2 M, PP3 Su) | - | X | - | 0.00874 | 0.00205 |
| C037* | ALL | 3 | Yes | CHEK2 | p.Thr410MetfsTer15 (3) (NM_001005735.2:c.1229del) | Het(86/96) | 0.90 | CIP | VUS (PVS1) | P (PVS1 VS, PP5 St, PM2 M, PP3 Su) | - | X | - | 0.00874 | 0.00205 |
| C124 | ALL | 15 | Yes | CHEK2 | p.Thr410MetfsTer15 (3) (NM_001005735.2:c.1229del) | Het(51/121) | 0.42 | CIP | VUS (PVS1) | P (PVS1 VS, PP5 St, PM2 M, PP3 Su) | - | X | - | 0.00874 | 0.00205 |
| C067 | ALL | 10 | Yes | FANCM | p.Arg1931Ter (6) (NM_020937.4:c.5791C>T) | Het(67/120) | 0.56 | P | P (PVS1, PP3, PP5) | P (PVS1 VS, PM2 M, PP3 Su) | (X) | X | - | 0.00448 | 0.00101 |
| C005 | CML | 14 | Yes | FANCM | p.Arg1931Ter (6) (NM_020937.4:c.5791C>T) | Het(113/220) | 0.51 | P | P (PVS1, PP3, PP5) | P (PVS1 VS, PM2 M, PP3 Su) | (X) | X | - | 0.00448 | 0.00101 |
| C143 | ALL | 11 | No | FANCM | p.Gln1701Ter (4) (NM_020937.4:c.5101C>T) | Het(48/111) | 0.43 | P / LP | VUS (PVS1, PP5) | P (PVS1 VS, PP5 VS, PM2 M, BP4 Su) | (X) | X | - | 0.00823 | 0.00129 |
| C108 | ALL | 16 | No | FANCM | p.Gln1701Ter (4) (NM_020937.4:c.5101C>T) | Het(99/205) | 0.48 | P / LP | VUS (PVS1, PP5) | P (PVS1 VS, PP5 VS, PM2 M, BP4 Su) | (X) | X | - | 0.00823 | 0.00129 |
| C092* | AA | 9 | No | FANCM | p.Gln1701Ter (4) (NM_020937.4:c.5101C>T) | Het(92/164) | 0.56 | P / LP | VUS (PVS1, PP5) | P (PVS1 VS, PP5 VS, PM2 M, BP4 Su) | (X) | X | - | 0.00823 | 0.00129 |
| C010* | Other (ALD) | 5 | No | HOXB13 | p.Gly84Glu (7)  (NM_006361.6:c.251G>A) | Het(194/363) | 0.53 | P / LP | VUS (PM1, PP2, PP3, PP5) | VUS (PP5 St, PP3 Su) | - | X | - | 0.00783 | 0.00186 |
| C089 | AML | 4 | Yes | MITF | p.Glu419Lys (NM_198159.3:c.1255G>A) | Het(128/263) | 0.49 | P / LP | LP (PM1, PM2, PP3, PP5, BS2) | P (PP5 VS, PS3 St, PM1 M, PP2 Su, PP3 Su) | - | X | - | 0.00111 | 0.00128 |
| C090 | AML | 4 | Yes | MSH6 | p.Phe1088SerfsTer2 (NM_000179.3:c.3261del) | Het(109/233) | 0.47 | P | P (PVS1, PM2, PP5) | P (PVS1 VS, PP5- VS, PS3 St, PP3 Su) | (X) | X | X | 0 | 0.00001 |
| C110 | ALL | 10 | No | MUTYH | p.Gly396Asp (NM_001128425.2:c.1187G>A) | Het(164/353) | 0.46 | P / LP | LP (PM1, PM2, PP3, PP5, BP1) | P (PP5 VS, PS3 St, PP3 St, PM1 M, PM5 M, PP2 Su) | - | X | X | 0.0022 | 0.00295 |
|  |  |  |  | MUTYH | p.Tyr179Cys (NM_001128425.2:c.536A>G) | Het(118/250) | 0.47 | P / LP | LP (PM1, PM2, PP3, PP5, BP1) | P (PP5 VS, PM1 M, PM2 M, PP2 Su, PP3 Su) | - | X | X | 0.00153 | 0.00154 |
| C034 | ALL | 1 | Yes | PMS2 | p.Glu109GlyfsTer30 (NM_000535.7:c.325dup) | Het(45/81) | 0.56 | P | P (PVS1, PM2, PP5) | P (PVS1 VS, PM2 M, PP3 Su) | (X) | X | X | 0 | 0.00002 |
| C094 | ALL | 0 | Yes | SDHC | p.His127Arg  (NM_003001.5:c.380A>G) | Het(93/196) | 0.47 | P / LP | LP (PM1, PM2, PP3, PP5) | P (PP5 St, PM1 M, PM2 M, PM5 M, PP3 Su, BP1 Su) | - | X | X | 0 | 0 |
| C041 | MDS | 2 | Yes | HFE | p.Cys282Tyr  (NM_000410.4:c.845G>A) | Hom(246/246) | 1.00 | CIP | LP (PS4, PM1, PP3, BS1) | P (PS1 St, PM1 M, PP5 M, PP2 Su, PP3 Su) | - | - | X | 0.03492 | 0.03238 |
| C152* | ALL | 5 | No | HFE | p.Cys282Tyr  (NM_000410.4:c.845G>A) | Hom(227/227) | 1.00 | CIP | LP (PS4, PM1, PP3, BS1) | P (PS1 St, PM1 M, PP5 M, PP2 Su, PP3 Su) | - | - | X | 0.03492 | 0.03238 |
| C011 | ALL | 5 | Yes | OTC | p.Arg40His  (NM_000531.6:c.119G>A) | Het(38/80) | 0.47 | P | LP (PM1, PM2, PP3, PP5) | P (PP5 VS, PM1 St, PM2 M, PM5 M, PP2 Su, PP3 Su) | - | - | X | 0 | 0 |
| C001 | CML | 10 | No | TTN | p.Pro26906GlnfsTer12 (NM_001256850.1:c.80717_80729del) | Het(171/334) | 0.51 | LP | P (PVS1, PM2, PP5) | P (PVS1 VS, PM2 M, PP5 M, PP3 Su) | - | - | X | 0.00005 | 0.00001 |
| C077 | ALL | 7 | Yes | TTN | p.Glu32813AsnfsTer3 (NM_001256850.1:c.98437del) | Het(137/329) | 0.42 | P / LP | P (PVS1, PM2, PP5) | P (PVS1 VS, PP5 St, PP3 Su) | - | - | X | 0.00005 | 0.00002 |
| C084* | BMF | 6 | No | TTN | p.Asn13247MetfsTer48 (NM_001256850.1:c.39740del) | Het(121/255) | 0.47 | LP | P (PVS1, PM2, PP5) | P (PVS1 VS, PM2 M, PP3 Su, PP5 Su) | - | - | X | 0 | 0 |

# Supplemental Figure 1. Kaplan-Meier survival curves for adult AML patients (n = 65) with and without harmful germline variant. A) Hematology Panel, B) Oncology Panel, C) ACMG SF 3.0 Panel, D) All Panels combined. The Log-rank test was used to calculate P-values.

## Supplemental Figure 1A. Hematology Panel. Survival curve of adult AML patients with, n= 3, and without, n= 62, at least one harmful germline variant in Hematology Panel genes.


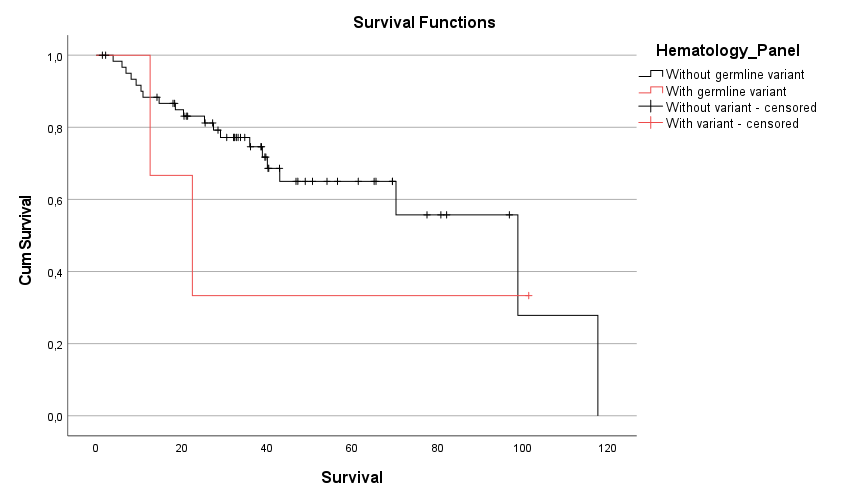


P _log-rank_ = 0.475

##

## Supplemental Figure 1B. Oncology Panel. Survival curve of adult AML patients with, n= 10, and without, n= 55, at least one harmful germline variant in Oncology Panel genes.


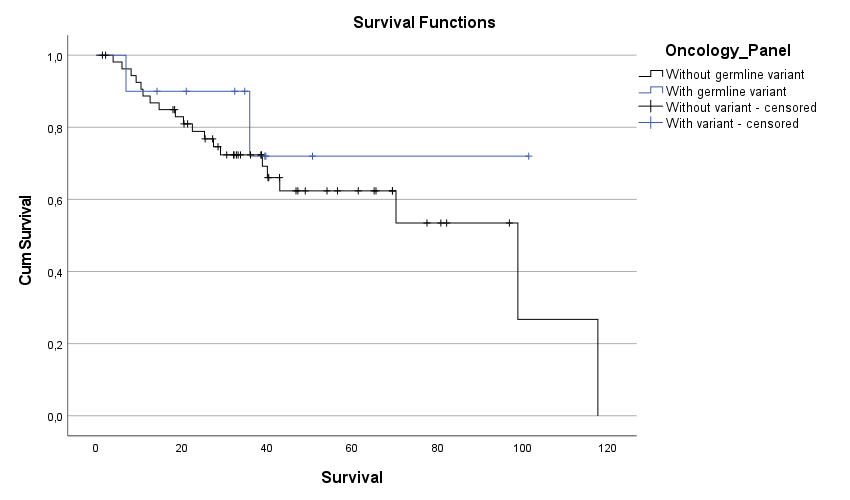


P _log-rank_ = 0.425

## Supplemental Figure 1C. ACMG SF v3.0 Panel. Survival curve of adult AML patients with, n= 1, and without, n= 64, at least one harmful germline variant in ACMG SF v3.0 Panel genes.


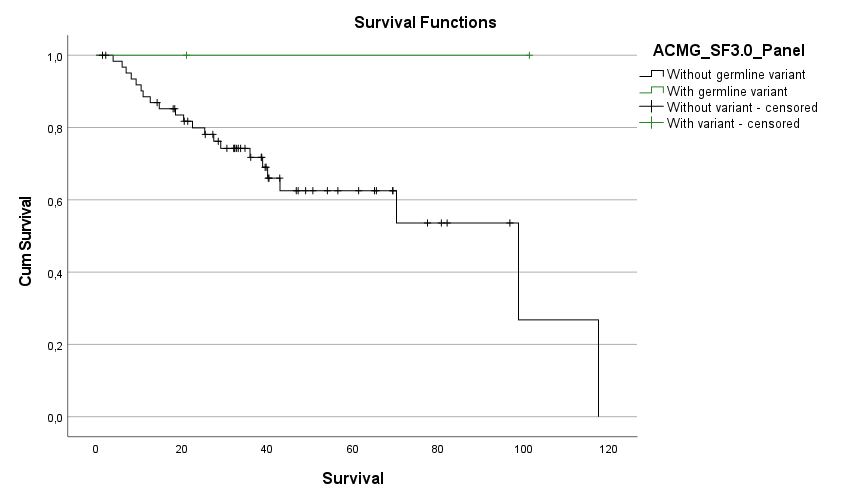


P _log-rank_ = 0.260

## Supplemental Figure 1D. All Panels. Survival curve of adult AML patients with, n= 12, and without, n= 53, at least one harmful germline variant in Hematology, Oncology or ACMG SF v3.0 Panel genes.


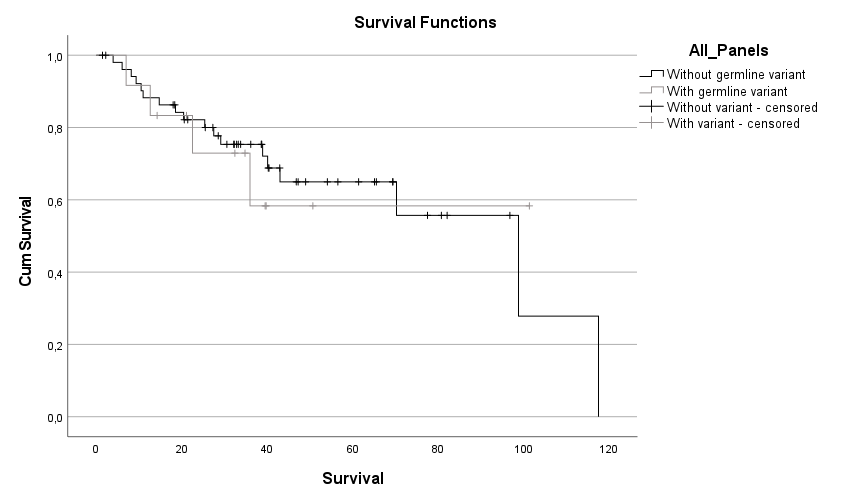


P _log-rank_ = 0.851

# References

1. Anand Pathak, Katja Seipel, Alexander Pemov, Ramita Dewan, Christina Brown, Sarangan Ravichandran, et al. Whole exome sequencing reveals a C-terminal germline variant in CEBPA-associated acute myeloid leukemia: 45-year follow up of a large family. Haematologica. 2016 Jul 1;101(7):846–52.

2. Douglas SPM, Siipola P, Kovanen PE, Pyörälä M, Kakko S, Savolainen E-R, et al. ERCC6L2 defines a novel entity within inherited acute myeloid leukemia. Blood. 2019 Jun 20;133(25):2724–8.

3. Näslund-Koch C, Nordestgaard BG, Bojesen SE. Increased Risk for Other Cancers in Addition to Breast Cancer for CHEK2*1100delC Heterozygotes Estimated From the Copenhagen General Population Study. J Clin Oncol. 2016 Feb 16;34(11):1208–16.

4. Kiiski JI, Pelttari LM, Khan S, Freysteinsdottir ES, Reynisdottir I, Hart SN, et al. Exome sequencing identifies FANCM as a susceptibility gene for triple-negative breast cancer. Proc Natl Acad Sci U S A. 2014 Oct;111(42):15172–7.

5. Nguyen-Dumont T, Myszka A, Karpinski P, Sasiadek MM, Akopyan H, Hammet F, et al. FANCM and RECQL genetic variants and breast cancer susceptibility: relevance to South Poland and West Ukraine. BMC Med Genet. 2018 Jan;19(1):12.

6. Peterlongo P, Catucci I, Colombo M, Caleca L, Mucaki E, Bogliolo M, et al. FANCM c.5791C>T nonsense mutation (rs144567652) induces exon skipping, affects DNA repair activity and is a familial breast cancer risk factor. Hum Mol Genet. 2015 Sep;24(18):5345–55.

7. Cannon-Albright LA, Stevens J, Teerlink CC, Agarwal N. The HOXB13 p.Gly84Glu variant observed in an extended five generation high-risk prostate cancer pedigree supports risk association for multiple cancer sites. Cancer Epidemiol. 2020;69:101834.

8. Meyts I, Aksentijevich I. Deficiency of Adenosine Deaminase 2 (DADA2): Updates on the Phenotype, Genetics, Pathogenesis, and Treatment. J Clin Immunol. 2018;38(5):569–78.

9. Cabral-Marques O, Klaver S, Schimke LF, Ascendino ÉH, Khan TA, Pereira PVS, et al. First Report of the Hyper-IgM Syndrome Registry of the Latin American Society for Immunodeficiencies: Novel Mutations, Unique Infections, and Outcomes. J Clin Immunol. 2014;34(2):146–56.

10. Jiang J, Tang W, An Y, Tang M, Wu J, Qin T, et al. Molecular and immunological characterization of DNA ligase IV deficiency. Clin Immunol. 2016;163:75–83.
